# Digitization of natural objects with micro CT and photographs

**DOI:** 10.1371/journal.pone.0195852

**Published:** 2018-04-12

**Authors:** Takashi Ijiri, Hideki Todo, Akira Hirabayashi, Kenji Kohiyama, Yoshinori Dobashi

**Affiliations:** 1 College of Engineering, Shibaura Institute of Technology, Toyosu, Tokyo, Japan; 2 Faculty of Liberal Arts, Chuo Gakuin University, Abiko, Chiba, Japan; 3 Graduate School of Information Science and Engineering, Ritsumeikan University, Kusatsu, Shiga, Japan; 4 Faculty of Environment and Information Studies, Keio University, Fujisawa, Kanagawa, Japan; 5 Graduate School of Information Science and Technology, Hokkaido University, Sapporo, Hokkaido, Japan; Chongqing University, CHINA

## Abstract

In this paper, we present a three-dimensional (3D) digitization technique for natural objects, such as insects and plants. The key idea is to combine X-ray computed tomography (CT) and photographs to obtain both complicated 3D shapes and surface textures of target specimens. We measure a specimen by using an X-ray CT device and a digital camera to obtain a CT volumetric image (volume) and multiple photographs. We then reconstruct a 3D model by segmenting the CT volume and generate a texture by projecting the photographs onto the model. To achieve this reconstruction, we introduce a technique for estimating a camera position for each photograph. We also present techniques for merging multiple textures generated from multiple photographs and recovering missing texture areas caused by occlusion. We illustrate the feasibility of our 3D digitization technique by digitizing 3D textured models of insects and flowers. The combination of X-ray CT and a digital camera makes it possible to successfully digitize specimens with complicated 3D structures accurately and allows us to browse both surface colors and internal structures.

## Introduction

Digitization is a process for converting a target specimen into a digital format. Natural objects, such as insects and plants, have been important targets of digitization because digital formats have various benefits, e.g., they are deterioration-free, space-efficient, and highly accessible. In addition, it is possible to capture and store digital information that is invisible to the naked eye by using various devices, e.g., highly detailed surface textures obtained with microscopy and internal structures captured with X-ray computed tomography (CT). Various digitization methods have been developed, and they store specimens with different data formats, such as two-dimensional (2D) photographs [1–3] or three-dimensional (3D) surface models [4–14]. In this paper, we also present a 3D reconstruction technique for natural objects based on measurement.

Many methods for the 3D digitization of plant and insect specimens have been studied. While some researchers reconstruct a 3D shape by using multiple photographs [4–11], other researchers adopt CT devices [12–14]. Both approaches have advantages and disadvantages. The image-based approach reconstructs 3D shapes from photographs taken from multiple viewpoints by adopting visual hull or stereo vision methods. Since this approach uses images as input, it obtains both shapes and textures at the same time; however, it is difficult to reconstruct concave and occluded structures. The CT-based approach takes an X-ray CT scan of a specimen to get a volumetric image (volume) and reconstructs the shape by segmenting the volume. This approach reconstructs a 3D shape accurately even if a specimen has concave and/or occluded structures. However, X-ray CT does not provide surface colors, and it is difficult to reconstruct surface textures.

In this paper, we present a novel digitization technique that combines image-based and CT-based approaches; we reconstruct a shape by using a CT volume of a specimen and generate a texture from photographs taken from multiple viewpoints. A key idea is camera position estimation; given a photograph and a 3D model obtained from a CT volume, we estimate the camera position of the photograph relative to the 3D model. We then project the photograph onto the 3D model from the found camera position to obtain a texture. We also provide techniques to combine multiple textures obtained from multiple photographs and to synthesize missing texture regions caused by occlusion.

We illustrate the feasibility of our technique by reconstructing 3D insect models and flower models with natural color textures. We evaluated the accuracy of our camera position estimation technique by artificially generating photographs of a 3D model with computer graphics and estimating their camera positions. Our technique was able to estimate camera position accurately. All data sets, including 3D models, textures, X-ray CT volumes, and photographs used in this paper, are available at the BioStudy repository (https://www.ebi.ac.uk/biostudies/studies/S-BSST121). Source codes are available at GitHub repository (https://github.com/TakashiIjiri/Digitization_pone) The same data and links are also available at the first author’s web page [15].

## Previous work

As we mentioned, the methods for digitizing insect or plant specimens are roughly divided into two approaches: image-based and CT-based approaches. We review both approaches below.

### Image-based approach

The image-based approach captures multiple photographs from different camera positions and reconstructs a 3D shape by adopting different methods such as shape-from-focus [4], structure-from-motion [5, 6], original multi-view-stereo [7], visual-hull [9], or structured-light [8, 10]. Existing methods were specialized to deal with different targets such as insects [4, 9], potted plants [5, 8], foliage [7], trees [6], and flowers [10]. Image-based approaches are able to obtain 3D shapes and surface colors at the same time. They, however, deal with only visible parts, and it is difficult for them to reconstruct the occluded structures that complicated insects and flowers often have.

### CT-based approach

To measure and store detailed information on specimens or to reconstruct the 3D shape of specimens with occluded structures, an X-ray CT device is adopted. Focusing on the potential of X-ray CT to produce quantitative volumes of biological samples, Metscher [12] provided easy-to-use staining methods and illustrated their feasibility by showing CT volumes of several fishes and insects. Faulwetter et al. [13] also explored the potential of X-ray CT as a new tool for supporting systematics and taxonomy. They discussed a way to build virtual type materials, named *cybertypes*, by X-ray CT and a way to use them. Ijiri et al. [14] adopted X-ray CT to reconstruct the 3D shapes of flowers that have highly occluded structures. They capture a sample flower by X-ray CT to obtain a volume and segment it into flower elements such as pistils, stamens, petals, and sepals by using a specially tailored algorithm.

X-ray CT captures internal structures, which are useful for scientific studies and for reconstructing objects with highly occluded structures. However, it does not capture the surface appearance; thus, it is difficult to reconstruct surface colors and textures from CT volumes.

### Combination of image-based and CT-based approach

3D modeling techniques that combine image-based and CT-based approaches have not been studied very much. Zhao et al. adopted such a combination to build volumetric appearance models of fabric [16]. They scan a small area of a fabric by micro CT and use the obtained CT volume to extract the orientation and density of fibers. They also take a photograph of the fabric to estimate optical appearance parameters. This method reconstructs an appearance model; however, it does not deal with 3D modeling.

## Methods

**Fig 1:**
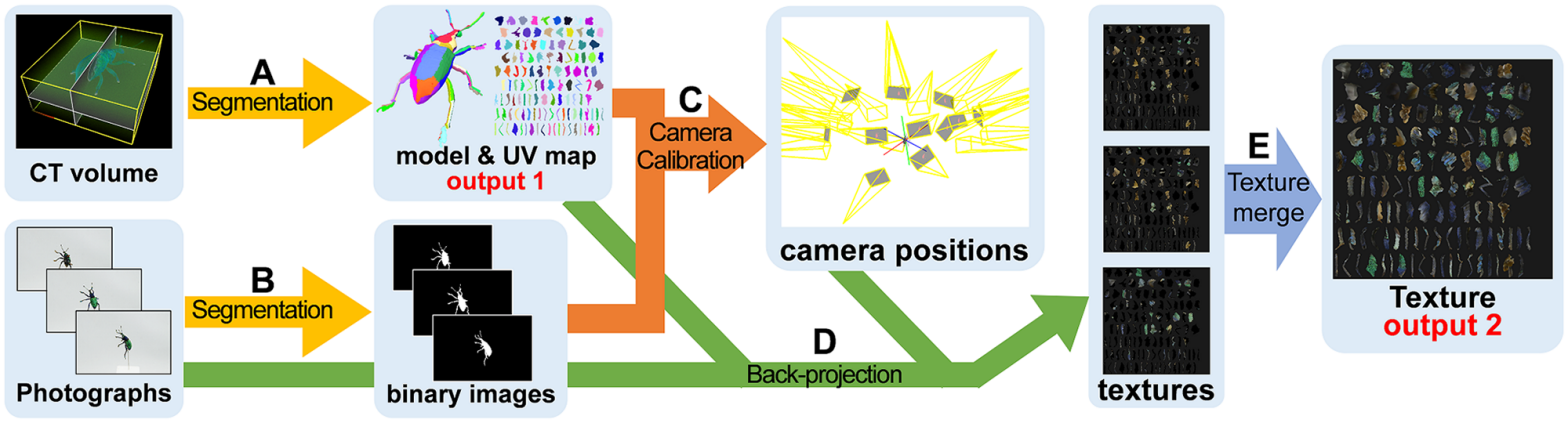
Overview of our digitization processes. Input CT volume is binarized to generate 3D model (A). Input photographs are also binarized (B). We estimate camera position relative to 3D model by using binarized photographs (C) and back project all photographs onto 3D model to obtain texture atlases (D). All texture atlases are merged to generate single texture (E).

We illustrate the workflow of our digitization process in Fig 1. To reconstruct textured 3D models of natural objects, we capture a specimen with an X-ray CT device and a digital camera. The obtained CT volume is semi-automatically segmented to reconstruct a 3D model and its UV map. We also semi-automatically segment the photographs taken with the camera. We next estimate the relative camera positions of the photographs by using their silhouettes. We then back project each photograph onto the 3D model to obtain a texture atlas. Since multiple photographs generate multiple textures, we merge them into a single texture atlas.

### Capturing X-ray CT and photographs

**Fig 2:**
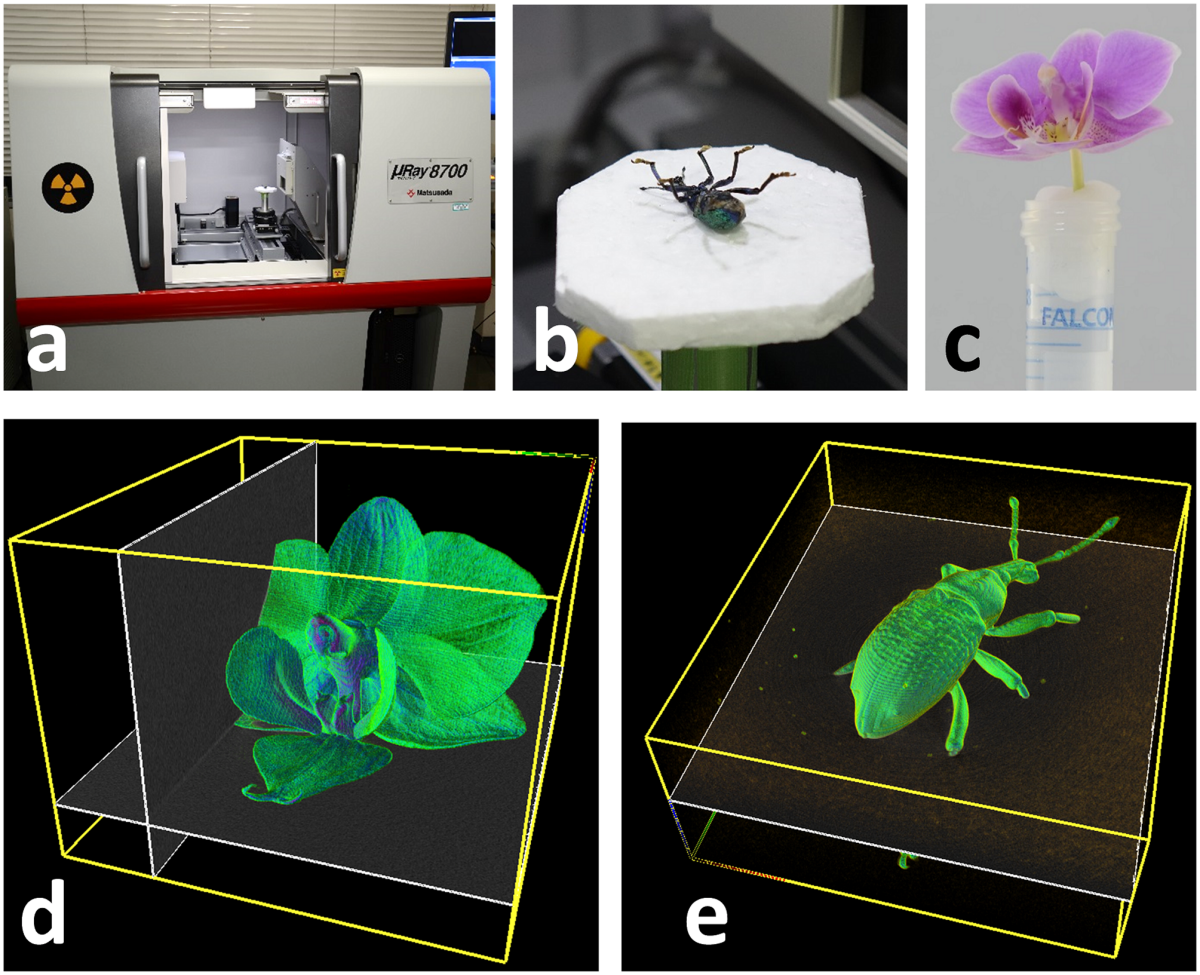
X-ray CT scanning. We used μRay8700 (a). We place insect directly on stage (b) and fix flower by using plastic tube (c). Obtained CT volumes are visualized in (d) and (e).

We first take an X-ray CT scan of a specimen. We use the Matsusada precision μRay8700 with a micro-focus X-ray tube (90-kV maximum voltage and 18-W maximum power), shown in Fig 2(a). For insects, we place a specimen directly on a stage (Fig 2b). For flowers, we fix them to the top of a plastic tube (Fig 2c). Fig 2(d) and 2(e) show obtained CT volumes.

**Fig 3:**
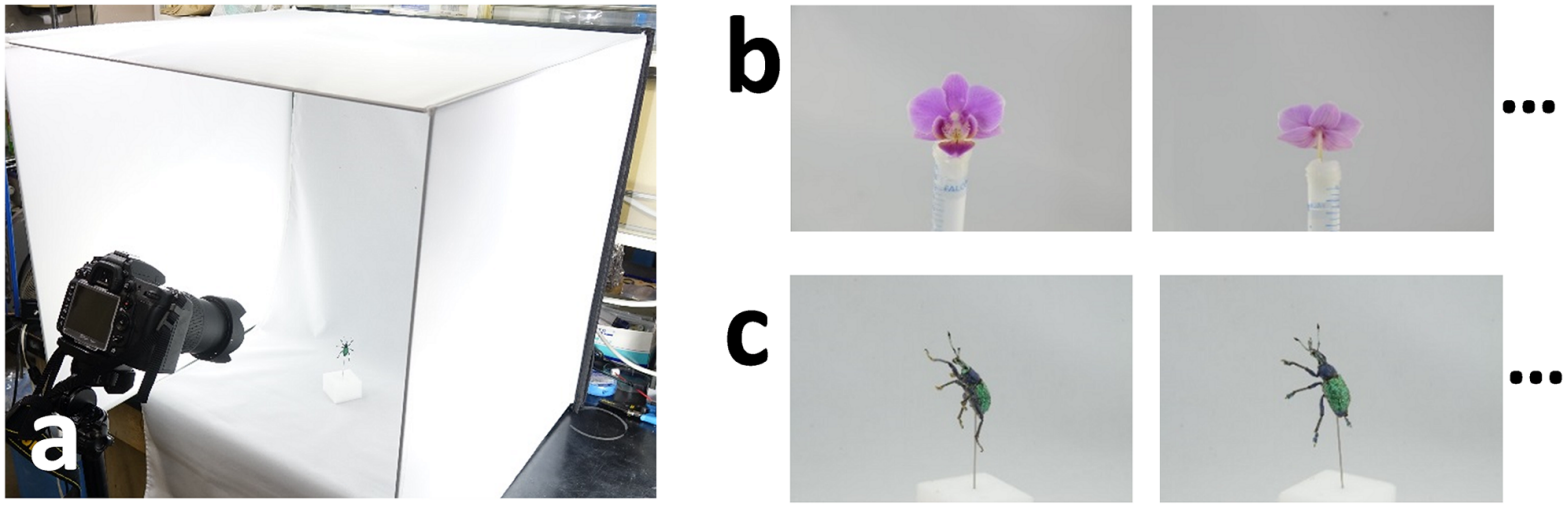
Photographing. We place specimen at center of light diffuser box (a) and take multiple photographs (b, c).

We next take multiple photographs from different viewpoints. We use a consumer grade digital camera, Nikon D7000, with a zoom lens, AF-S Nikkor 18–105 mm. We set up the lighting condition by using a fabric light diffuser to provide soft light, as shown in Fig 3(a). We place a specimen at the center of a diffuser box and take multiple photographs by manually rotating the specimen and modifying the height of the camera. When taking photographs, we do not store the accurate position of the camera relative to the specimen, which will be estimated in subsequent processes. Fig 3(b) and 3(c) show representative photographs. During photographing, we fixed an insect with a pin. We also semi-automatically binarized all of the photographs by adopting a graph-cut segmentation technique [17, 18].

### 3D surface model reconstruction

**Fig 4:**
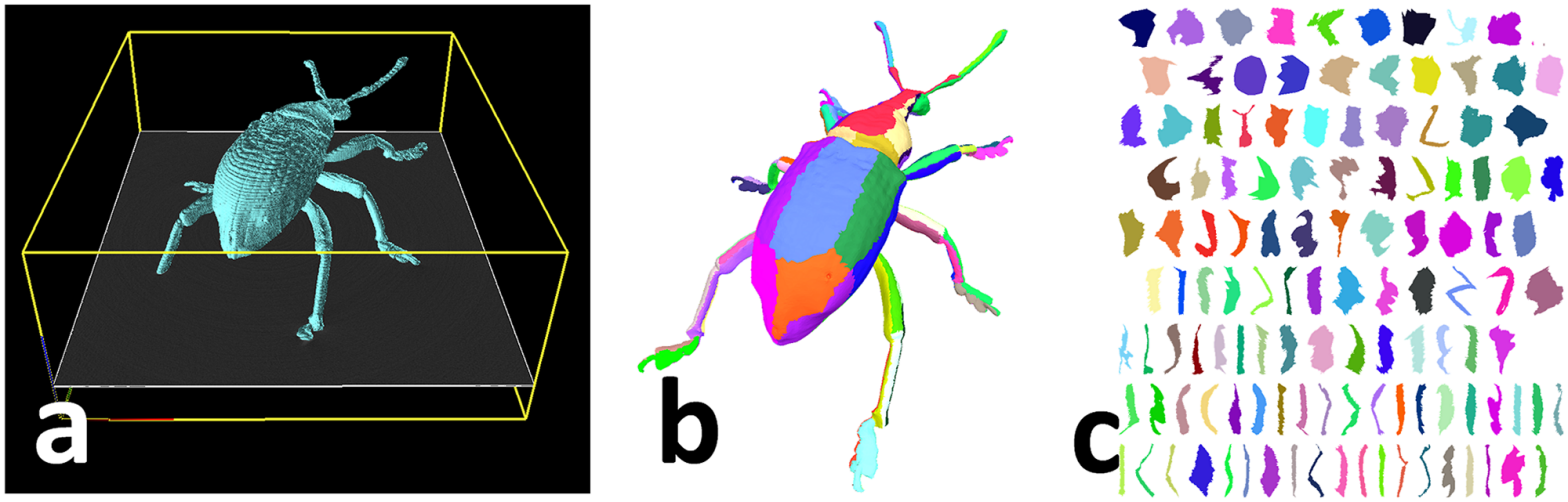
Surface reconstruction. We extracted foreground from input CT volume (a) to obtain surface model (b). We segmented surface into near-flat regions (b) and flattened each region into 2D texture domain (c).

With our setup, an obtained CT volume contains the target specimen and air only. We thus successfully extract foreground voxels by combining region growing with a threshold, morphological operations, i.e., opening and closing, and hollow region filling. For this purpose, we adopt our developed 3D volume processing software, RoiPainter3D [19]. We interactively tuned the threshold and the order of operations according to the targets. After segmentation, we adopt the marching cubes algorithm [20] to obtain a surface model (Fig 4a and 4b).

Since we focus on texture reconstruction from input photographs, additional 2D texture domain parametrization is required for the surface model. We first segment the model into near-flat regions by performing a distortion-aware region growing process [21] (Fig 4b). To build a complete texture atlas, we flatten each region by adopting the *Unfold3D* function of Autodesk Maya [22] (Fig 4c).

### Camera position estimation—Optimization definition

For each input photograph, we estimate a camera position relative to the 3D model. In other words, we would like to obtain a camera position from which the rendered image of the 3D model matches the photograph. Since the 3D model does not have a texture yet, only its shape is available. We thus perform silhouette matching; we search for a camera position that provides a rendered image of which the silhouette matches that of the photograph.

#### Camera representation

We adopt a simple pinhole camera projection model [23]. A point (*x*, *y*, *z*) in a 3D camera coordinate system is projected onto a 2D screen space as $\left(x^{\prime },\;y\prime )\;=\;(\frac{xf}{z},\frac{yf}{z}\right)$, where (*x*′, *y*′) is a projected 2D position and *f* is a focal length. We suppose that the focal length and sensor size used for photographing is given.

**Fig 5:**
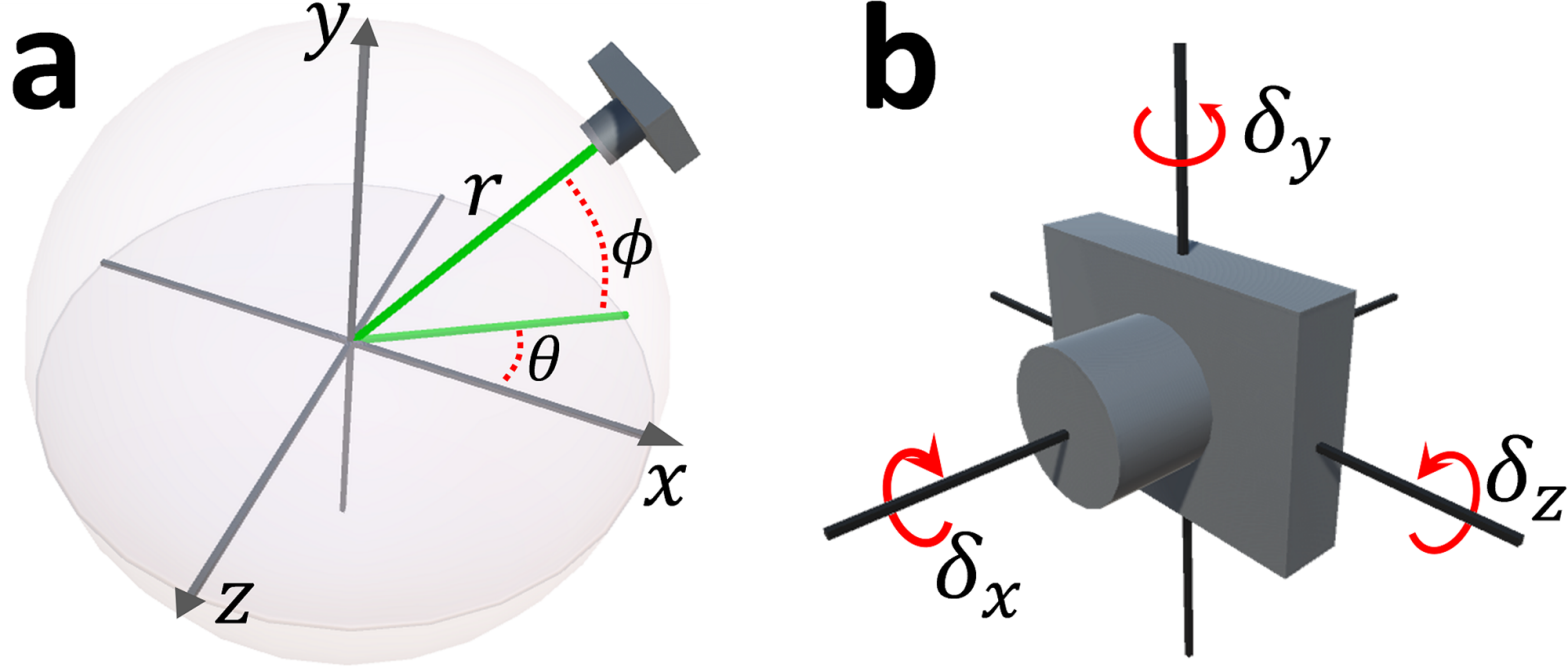
Representation of camera position.

We represent a local camera position with six parameters **c** = (*θ*, *ϕ*, *r*, *δ*_*x*_, *δ*_*y*_, *δ*_*z*_). Three values (*θ*, *ϕ*, *r*) indicate the position in a spherical polar coordinate system of which the origin is the mass center of the 3D model (Fig 5a). The other three values (*δ*_*x*_, *δ*_*y*_, *δ*_*z*_) indicate the local rotation of the camera (Fig 5b). From the six parameters, the position **c**_*pos*_ ∈ *R*^3^, ray direction **c**_*ray*_ ∈ *R*^3^, and up vector **c**_*up*_ ∈ *R*^3^ of the camera can be defined as

$$c_{pos}=R_{\theta }^{y}R_{\phi }^{z}\left(\begin{array}{c} r\\ 0\\ 0 \end{array}\right),c_{ray}=R_{\theta }^{y}R_{\phi }^{z}R_{\delta_{x}}^{x}R_{\delta_{y}}^{y}R_{\delta_{z}}^{z}\left(\begin{array}{c} -1\\ 0\\ 0 \end{array}\right),c_{up}=R_{\theta }^{y}R_{\phi }^{z}R_{\delta_{x}}^{x}R_{\delta_{y}}^{y}R_{\delta_{z}}^{z}\left(\begin{array}{c} 0\\ 1\\ 0 \end{array}\right)$$ (1)

where $R_{\beta }^{\alpha }\in R^{3\times 3\;}$ is a rotation matrix along an axis *α* with an angle *β*. The set of three vectors (**c**_*pos*_, **c**_*ray*_, **c**_*up*_) determines a unique camera coordinate system.

#### Optimization problem

**Fig 6:**
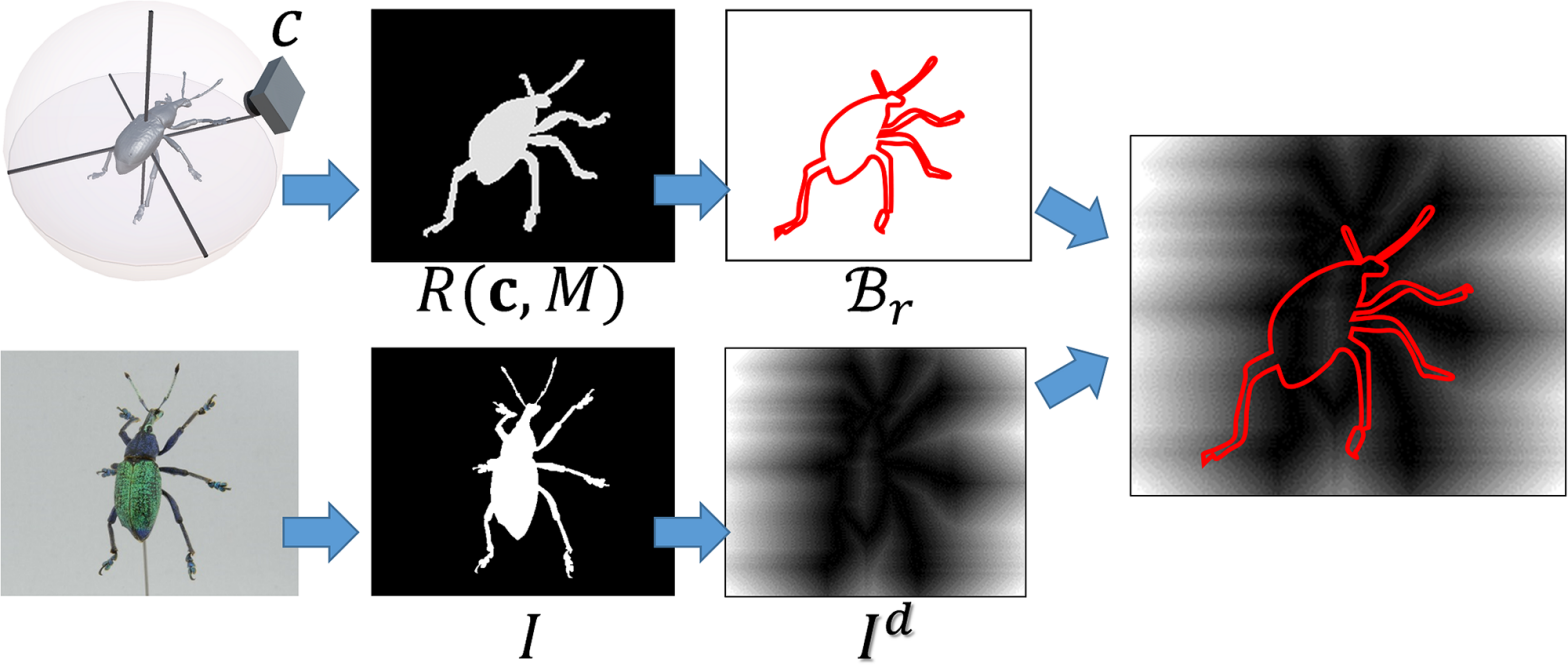
Difference metric of two binary images.

Given a 3D model *M* and a binarized photograph *I*, we estimate a camera position c* from which the rendered image of *M* matches *I*. This can be formulated as the following minimization problem,

$$c^{*}\;=\;{argmin}_{c}\;d(I,\;R(c,\;M)),$$ (2)

where *R*(**c**, *M*) is a binarized rendered image of *M* from the camera position **c** and d(*I*, *R*(**c**, *M*)) is a metric for measuring the difference between two binary images *I* and *R*(**c**, *M*). According to [24], the difference metric is defined as

$$d(I,\;R(c,\;M))\;=\;\frac{1}{\left|B_{r}\right|}\sum_{p\in B_{r}}I^{d}(p),$$ (3)

where *I*^*d*^ is a distance transform image of *I*, *B*_*r*_ is a set of boundary pixels of *R*(**c**, *M*), and |*B*_*r*_| is the number of the boundary pixels. Fig 6 depicts this difference metric; it overlaps the boundary pixels *B*_*r*_ onto the distance transform image *I*^*d*^ and sums up the distance values under the boundary pixels. In other words, this metric provides an average distance between the boundaries of two binary images.

### Camera position estimation—Implementation

To solve the optimization problem in Eqs (2) and (3) efficiently, we present an algorithm that consists of three steps: (i) initial estimation by coarse exhaustive search, (ii) gradient descent optimization, and (iii) hill climbing.

#### Initial estimation

To obtain an initial camera parameter **c**^0^, we first perform a coarse exhaustive search by varying the three positional parameters (*θ*, *ϕ*, *r*). Specifically, we generate 3D points by near-regularly sampling a unit sphere and compute the azimuth *θ*_*k*_ and the altitude *ϕ*_*k*_ for each point to obtain the candidate angles (*θ*_*k*_, *ϕ*_*k*_). We suppose that an approximate distance *r*_0_ between the camera and a specimen during photographing is given and prepare a set of candidate distances *r*_*l*_ ∈ {*r*_0_, *r*_0_ ± 10, *r*_0_ ± 20, *r*_0_ ± 30}, where the distance is expressed in [mm] units. All combinations of the above angles and distances provide the candidates of camera parameters **c**_*i*_ = (*θ*_*i*_, *ϕ*_*i*_, *r*_*i*_, 0,0,0).

**Fig 7:**
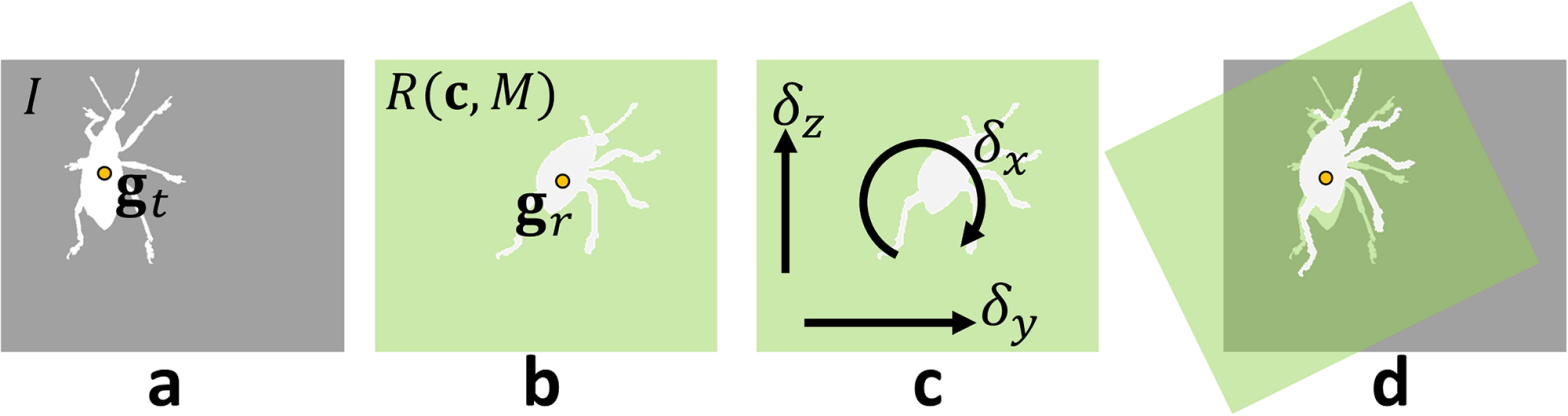
Effect of camera rotation. Camera rotations represented with *δ*_*x*_, *δ*_*y*_, and *δ*_*z*_ correspond to rotation, horizontal translation, and vertical translation of rendered image, respectively (c). In Eq (4), we rotate and translate rendered image (b) to fit it to photograph (d).

We then generate multiple rendered images of *M* from all of the candidates. Since the camera rotation angles (*δ*_*x*_, *δ*_*y*_, *δ*_*z*_) of **c**_*i*_ are set to be zero, a rendered image *R*(**c**_*i*_, *M*) does not include camera rotation and cannot be used to evaluate the metric (3) as it is. However, if we build candidates **c**_*i*_ with a variation in rotation angles (*δ*_*x*_, *δ*_*y*_, *δ*_*z*_), the combination explodes. Instead, we mimic camera rotation by translating and rotating a 2D rendered image. Specifically, we modify the metric (3) to consider the best fitting 2D translation and rotation as

$$d^{\prime }(I,\;R(c_{i},\;M))\;=\;\underset{\alpha \;}{\text{min}}\;\sum_{p\in B_{r}}I^{d}(R_{\alpha }(p-g_{r})+g_{t}),$$ (4)

where **R**_*α*_ ∈ **R**^2×2^ is a 2D rotation matrix with an angle *α* and **g**_*t*_ = (*g*_*tx*_, *g*_*ty*_) and **g**_*r*_ = (*g*_*rx*_, *g*_*ry*_) denote the mass centers of the foregrounds of *I* and *R*(**c**_*i*_, *M*), respectively (Fig 7). The metric (4) includes a minimization problem in itself; we solve it with coarse exhaustive search, where we vary *α* ∈ [0°, 360°] with an interval of 1°.

The exhaustive search described above finds the initial camera position (*θ*^0^, *ϕ*^0^, *r*^0^) and 2D rotation angle *α*. The initial camera rotation angles $\left(\delta_{x}^{0},\delta_{y}^{0},\delta_{z}^{0}\right)$ can also be computed as

$$\delta_{x}\;=\;-\alpha ,\;\delta_{y}\;=\;{\text{tan}}^{-1}\;\frac{-g_{rx}^{\prime }+g_{tx}}{f},\;\delta_{z}\;=\;{\text{tan}}^{-1}\;\frac{-g_{ry}^{\prime }+g_{ty}}{f},$$ (5)

where $(g_{rx}^{\prime },g_{ry}^{\prime })\;=\;R_{\alpha }g_{r}$ and *f* is a focal length used for both photographing and rendering. Note that *f*, **g**_*t*_, and **g**_*r*_ are represented in pixel units.

Although this exhaustive search tests a large amount of candidates, it finishes in a reasonable computational time. Notice that the distance transform of a photograph is precomputed only one time before the search. In addition, if multiple photographs have the same candidate distances, the generated rendered images can be shared for the photographs. In our experience, when we prepared 2,562 candidate angles, it took about a half minute to precompute 2,562 rendered images and about a few seconds for the exhaustive search.

#### Gradient decent iteration

Given an initial camera parameter $c^{0}\;=\;(\theta^{0},\;\phi^{0},\;r^{0},\;\delta_{x}^{0},\delta_{y}^{0},\delta_{z}^{0})$, we iteratively update it by using a gradient descent method as

$$c^{t+1}\;=\;c^{t}-h\;s\circ \nabla d(I,\;R(c^{t},\;M)),$$ (6)

where *t* is the number of iterations, *h* ∈ *R*^1^ is a step size which we determine by the line search strategy, ***s*** ∈ *R*^6^ is a scale coefficient, and ∘ represents the Hadamard product. We approximate the gradient ∇d(∙,∙) with the central difference. For instance, when computing the gradient with respect to the *k*-th dimension of **c**, we prepare two camera parameters **c**^*t*^ ± *h*_*k*_**e**_*k*_, where **e**_*k*_ is the *k*-th standard basis in *R*^6^ and *h*_*k*_ is an offset coefficient. We render two images from them *R*(**c**^*t*^ ± *h*_*k*_**e**_*k*_, *M*) and approximate the gradient as $\frac{\partial d(I,\;R(c^{t},\;M))}{\partial \theta_{k}}\;=\;\frac{d(I,\;R(c^{t}+h_{k}e_{k},\;M))-d(I,\;R(c^{t}-h_{k}e_{k},\;M))}{2\;h_{k}}$.

#### Hill climbing

The gradient descent iteration may fall into local minima. To avoid this issue, we additionally perform a random walk based method, i.e., the hill climbing algorithm. Starting from the solution found at the gradient descent step, we iteratively update it; at each iteration step, we select an element (dimension) of the current solution **c**, modify the element by adding a random offset to obtain a new parameter **c**′, and accept **c**′ if **c**′ improves the difference metric. We repeat this process for a fixed number of times to obtain a final camera parameter c*.

#### Implementation details

To compute the distance metric (3) efficiently, we reduce the sizes of input photographs to 1/8 and generated rendered images so that they had the same size. In this study, photographs with 3696 × 2448 pixels were reduced to 462 × 306 pixels. For the initial estimation by exhaustive search, we prepared 2,562 candidate angles by uniformly sampling a unit sphere. For the gradient descent iteration, we empirically selected the following parameters: **s** = (10^−4^, 10^−4^, 10^1^, 10^−7^, 10^−7^, 10^−7^), $h_{1}\;=\;h_{2}\;=\;h_{4}\;=\;h_{5}\;=\;h_{6}\;=\;0.1\times \frac{2\pi }{360}$, *h*_3_ = 10. For the hill climbing, we modified an element of the current parameter **c** by adding a random value sampled from [−0.1, 0.1] for the 2nd dimension (*r*) and a value sampled from [−10^−2^, 10^2^] for the other dimensions (*θ*, *ϕ*, δ_*x*_, *δ*_*y*_, *δ*_*z*_). We performed the hill climbing iterations 1000 times. Also, we discard input photographs of which the difference metric (3) is not less than a threshold (we used 0.7 in this study) and do not use such photographs in the following process since our algorithm fails to find their camera positions properly.

### Texture reconstruction

**Fig 8:**
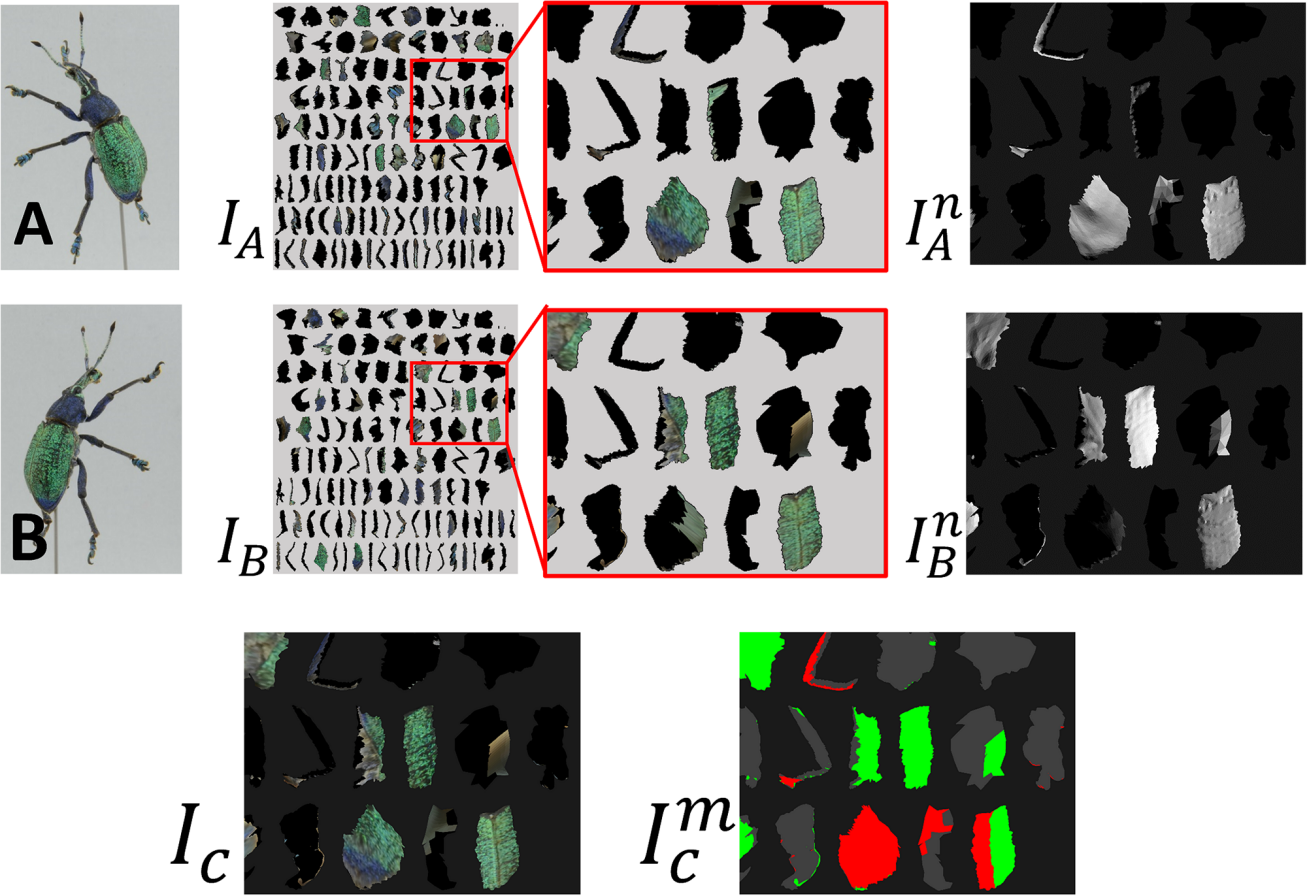
Texture generation. Each photograph (A and B) generates texture atlas (*I*_*A*_ and *I*_*B*_). We stitch two textures with indistinct seam to obtain one texture (*I*_*C*_). To obtain clearer texture, we consider both texture color and projected normal direction ($I_{A}^{n}$ and $I_{B}^{n}).\;I_{C}^{m}$ visualizes used texture with different color (red for A and green for B).

In this Subsection, we reconstruct a single texture by stitching multiple textures generated from multiple photographs. Given a camera parameter for each input photograph, we back project a photograph onto a 3D model to generate a texture atlas. In Fig 8, the input photographs (A) and (B) are back projected onto a 3D model to generate texture atlases (*I*_*A*_) and (*I*_*B*_). Since multiple photographs usually cover the same area, it is necessary to stich textures without distinct seams. For this purpose, we present an extension of graph-cut texture synthesis [25]. We first introduce a technique for stitching *two* textures and then discuss a method for dealing with multiple textures.

#### Stitching two textures with graph cut [25]

**Fig 9:**
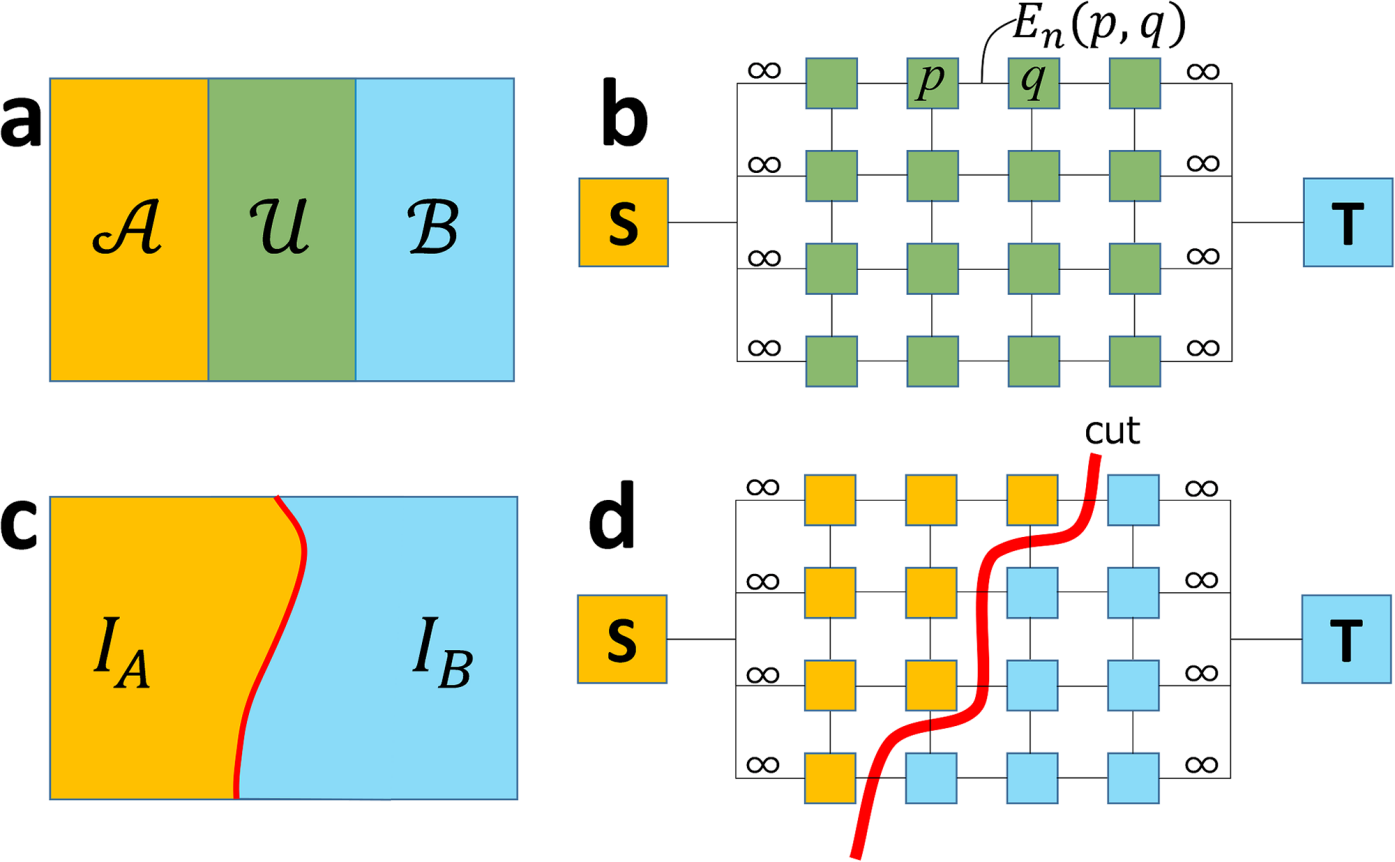
Graph-cut textures to stitch two textures with indistinctive seam.

When stitching together two textures *I*_*A*_ and *I*_*B*_, a texture can be classified into three regions, A, B, and U. Region A is covered only with *I*_*A*_, B is covered only with *I*_*B*_, and U is covered with both *I*_*A*_ and *I*_*B*_. As shown in Fig 9, we compute a seam in region U by using the graph-cut method. As shown in Fig 9(b), we construct a graph such that its nodes consist of pixels in U, the source node *S*, and the sink node *T*. Each pixel node *p* is connected to its neighbors *q* with an edge. The edge capacity *E*_*n*_(*p*, *q*) is defined as

$$E_{n}(p,q)\;=\;|I_{A}(p)-I_{B}(q)|-\;|I_{A}(p)-I_{B}(q)|.$$ (7)

The pixel nodes neighboring region A are connected to *S*, and those neighboring B are connected to *T* with edges. Their capacities are ∞. We compute the minimum cut of this graph and use the cut as the seam to stitch *I*_*A*_ and *I*_*B*_ (Fig 9c and 9d).

#### Extension of graph-cut textures

**Fig 10:**
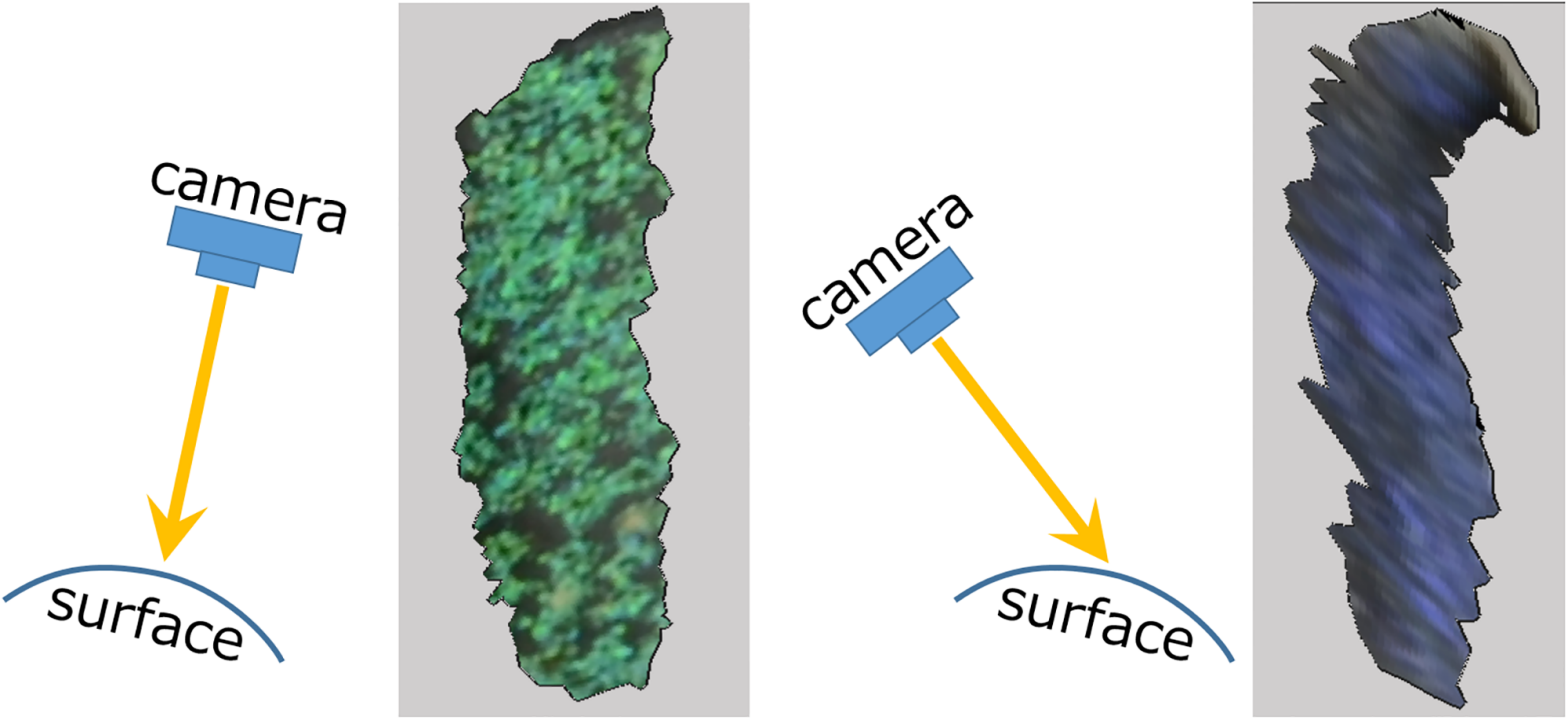
Blurred textures caused by back-projection.

Since textures are generated by back-projecting photographs onto a 3D model, a clear texture image is obtained around the region where the surface normal is oriented to the camera. A blurred texture is generated around the region where the surface normal is not oriented to the camera (Fig 10). When computing a seam, we prefer to use the texture region where the surface normal is oriented to the camera. To do this, we introduce a simple extension to the original graph-cut method [25]; we connect each pixel node *p* to source *S* and sink *T* with edges *E*(*p*, *S*) and *E*(*p*, *T*). Their capacities are defined as

$$E_{t}(p,S)\;=\;-I_{A}^{n}(p),\;E_{t}(p,T)\;=\;-I_{B}^{n}(p),$$ (8)

where $I_{A}^{n}(p)$ is a dot product of the camera ray and surface normal at pixel *p* when generating *I*_*A*_ and $I_{B}^{n}(p)$ is similarly defined. Note that $-I_{A}^{n}(p)$ becomes large if the surface normal at *p* is oriented to the camera. With this simple modification, we integrate two textures such that two images are stitched together with an indistinctive seam and clearer texture pixels are preferred.

The technique mentioned above is for combining two textures. When dealing with multiple textures, we adopt it multiple times. Given multiple textures, we first stitch two of them to obtain a resulting texture. We then select a non-combined texture and combine it into the resulting texture. We repeat this process until combining the all given textures.

### Occlusion reconstruction

**Fig 11:**
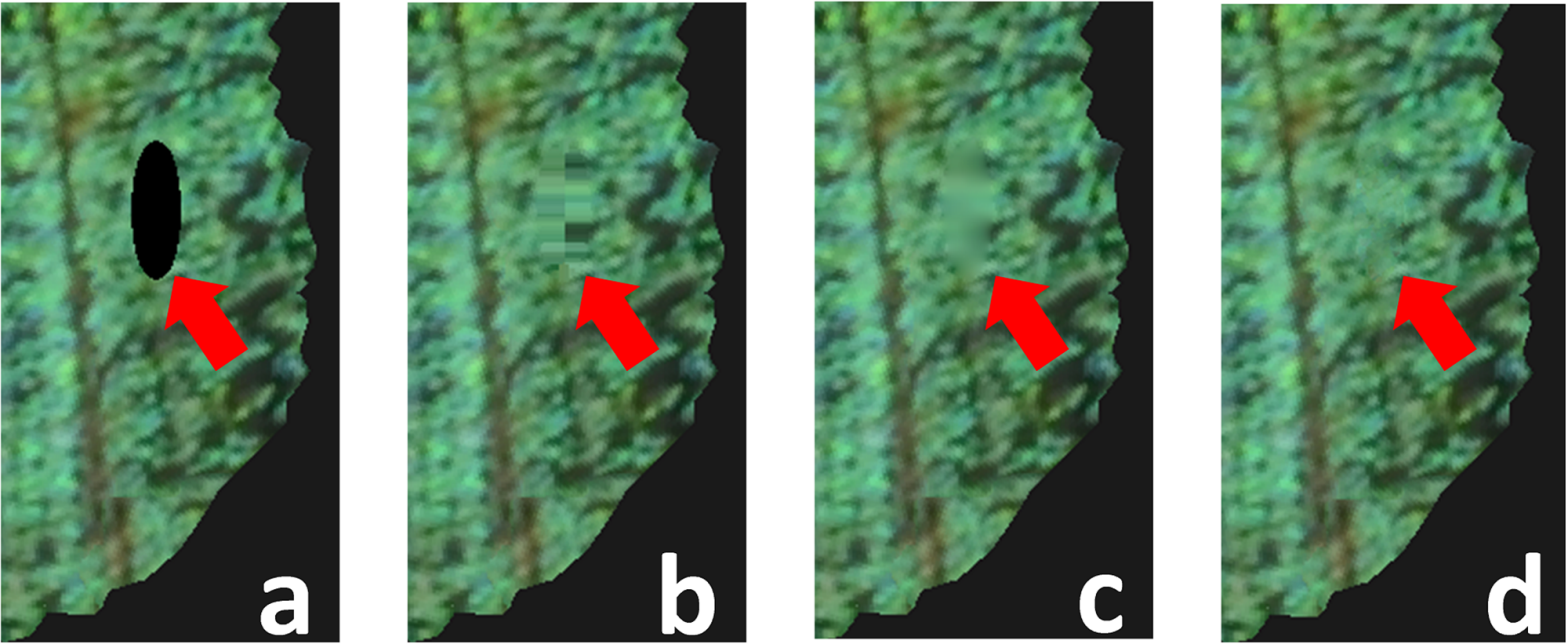
Patch-based texture synthesis to recover missing area. For black circular area (a) (artificially generated for explanation), we fill it in by copying boundary pixels (b), smoothen it (c) and perform texture synthesis to refine it (d).

Since we reconstruct a texture by back projecting multiple photographs, it is impossible to reconstruct texture areas hidden by occlusion. We recover such occluded region by copying boundary colors and performing texture synthesis. Fig 11 summarizes the process. For each missing area (a), we first fill in an area by iteratively growing the boundary and copying pixel colors from boundary (b). We next adopt simple smoothing to obtain a blurred texture in the area (c).

By adopting a texture synthesis method [26] presented in the graphics field, it is possible to further enhance the appearance of a missing area as in Fig 11(d). This is a straightforward extension of the texture synthesis algorithm [26] to a surface model. See algorithm 1 in the original paper for details [26]. We present only a summary of the process below.

We first prepare a large number of *reference patches* such that we randomly place points on a 3D model and generate a small texture patch for each point by sampling the texture color around the point. We adopt exponential mapping [27] for sampling texture colors on the model and discard patches that contain pixels of the missing area. Next, we randomly sample target pixels from the missing texture area and create a *target patch* around each of the target pixels. We also adopt exponential mapping in this process. For each target patch, we search the most similar reference patch and blend the found reference patch around the target pixel. We repeat this search and blend process several times to obtain the final results.

Notice that the texture synthesis described above generates a “fake” texture. It thus should be used only for computer graphics purposes and not for scientific digitization. To reconstruct textures in occluded areas correctly, Yin et al. [11] presented an intrusive method in which they excised leaves and captured their shapes and textures separately. To adopt such an intrusive method to our targets remains as future work.

## Results and discussions

### Accuracy of our camera estimation technique

**Fig 12:**
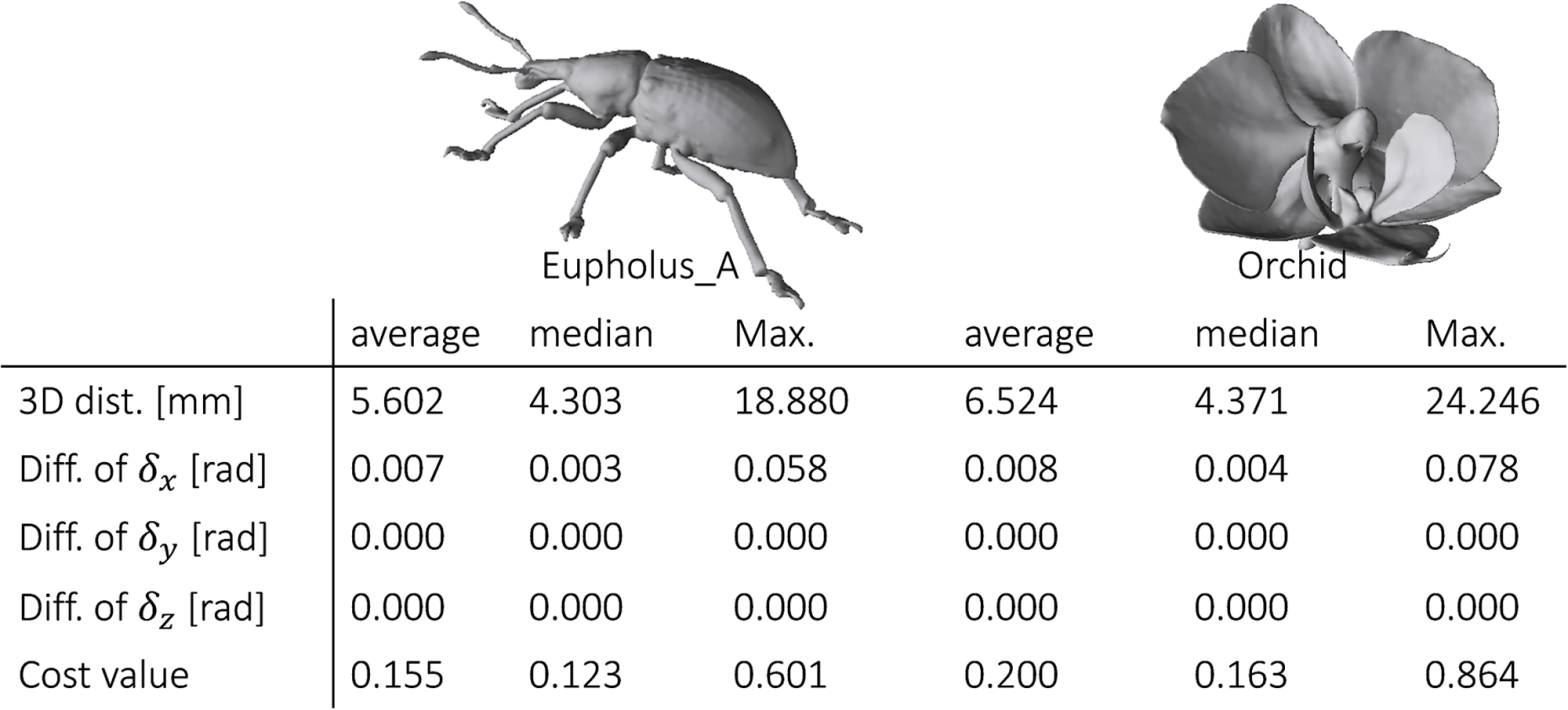
Evaluation of our camera estimation by using artificial data set. This figure shows average, median, and maximum camera estimation errors and cost values of our technique for artificially generated photographs.

We first evaluated the accuracy of our camera position estimation technique. We prepared two 3D models (*Eupholus_A* and *Orchid*, Fig 12 top) reconstructed from X-ray CT volumes. We also generated 100 camera positions by sampling each element of 6D parameters (*r*, θ, *ϕ*, δ_*x*_, *δ*_*y*_, *δ*_*z*_) from uniform random distributions; the range of each parameter was *r* ∈ [540, 560], *θ* ∈ [0,2*π*], $\phi \in [-\frac{0.7\pi }{2},\frac{0.7\pi }{2}]$, δ_*x*_ ∈ [0,2*π*], and *δ*_*y*_ ∈ [−0.006*π*, 0.006*π*], *δ*_*z*_ ∈ [−0.006*π*, 0.006*π*]. We then created rendered images of the two models from them. We estimated a camera position for each artificially generated rendered image by adopting our technique and evaluated the estimation error; we measured the distance between the estimated and the correct camera positions in 3D, the differences in rotation angles (δ_*x*_, *δ*_*y*_, *δ*_*z*_), and the cost value defined in Eq (3). The evaluated errors are summarized in Fig 12. The median of the distances was less than 5.0 mm. The median of the cost value was less than 0.2. Notice that the cost value represents the mean distance between the silhouette of an input image and that of a rendered image from the estimated camera position. Although our technique uses only the silhouette information of an input image, it is able to estimate camera positions accurately.

### Generated models

**Fig 13:**
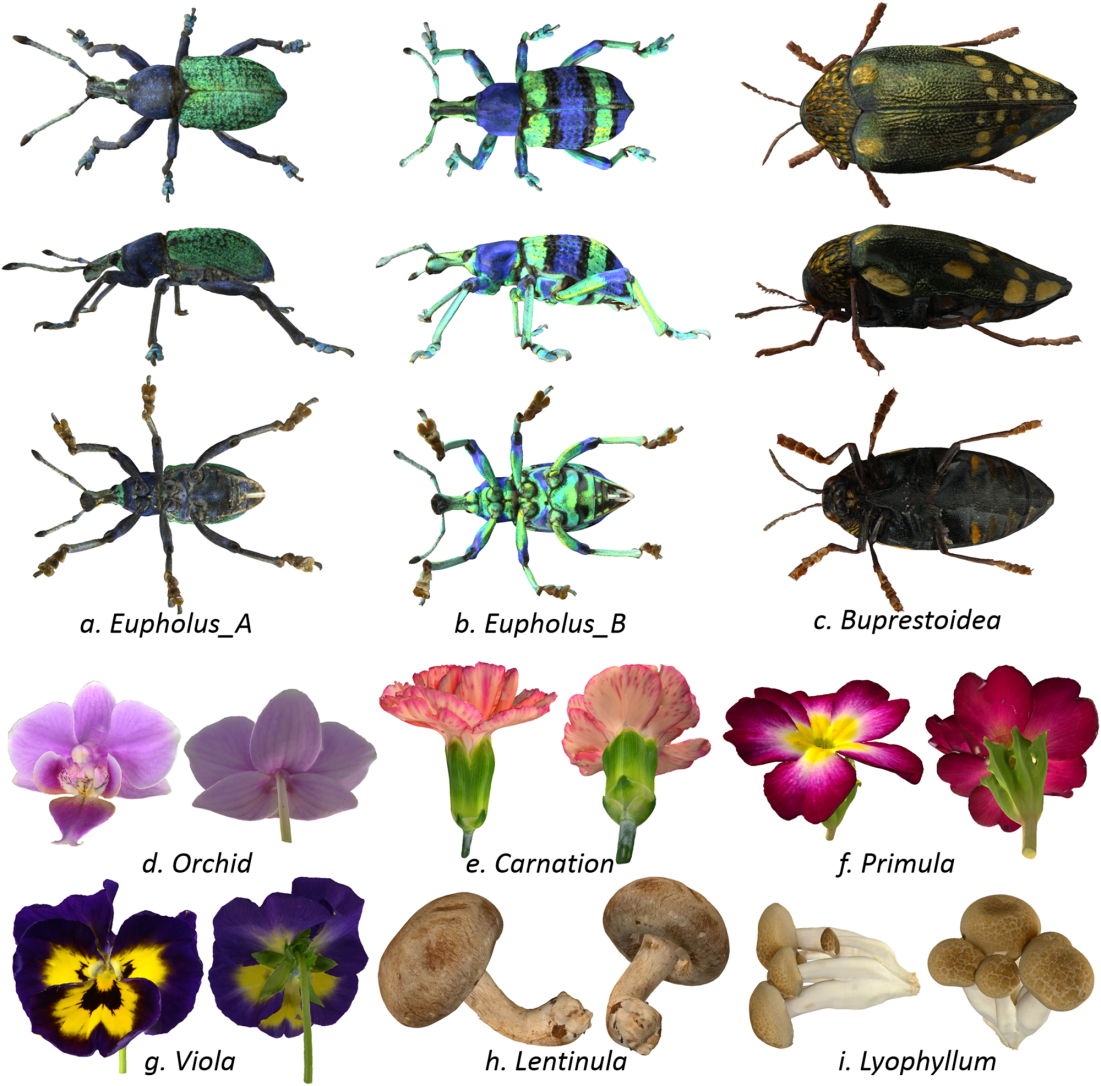
3D models generated with our technique.

Fig 13 shows the 3D models generated by our technique. We reconstructed models and textures from both X-ray CT volumes and photographs, which made it possible to digitize specimens with highly occluded structures, such as flowers. Since our technique reconstructs shapes from CT volumes, the number of required photographs was relatively small compared with pure image-based modeling methods, e.g., [9]. The resulting dataset are available on the first author’s web page [15].

**Fig 14:**
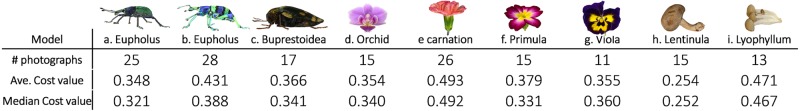
Detailed data for models in Fig 13. First row shows number of photographs used to reconstruct models in Fig 13. Second and third rows show average and median of cost values for camera position estimation in Eq (3). Notice that photographs of which matching cost were greater than or equal to 0.7 were not used for texture generation and not counted in this table.

Fig 14 shows the number of photographs used to reconstruct each model and the average/median/maximum matching cost values of the used photographs. Notice that we did not use photographs of which the matching costs were not less than 0.7 for texture reconstruction. The median of the cost values was less than 0.5. Our technique successfully estimates camera positions for real-world data sets.

**Fig 15:**
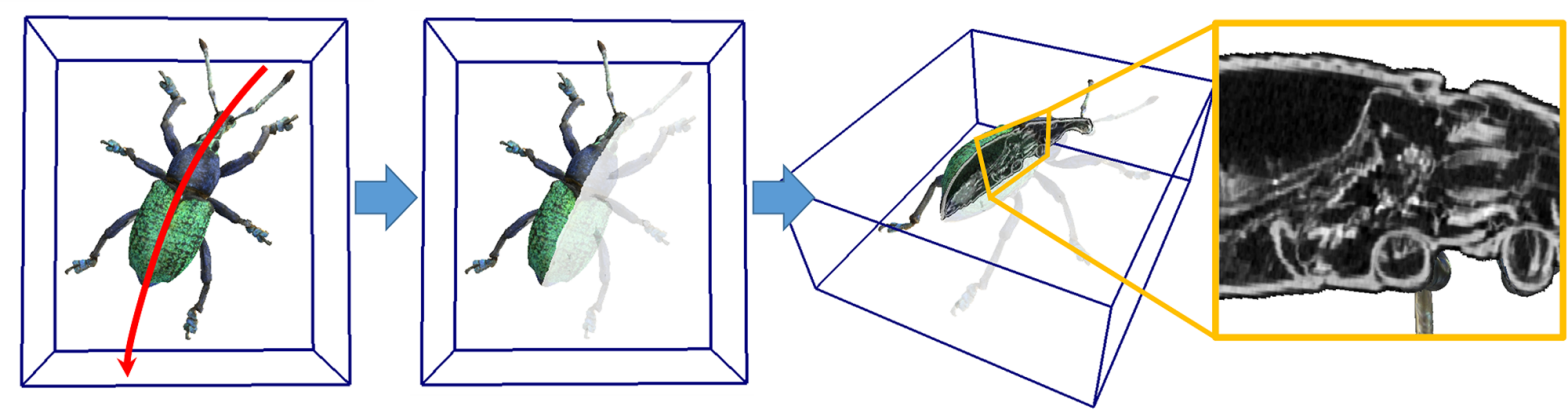
Cross-section visualization. Our browsing system allows user to draw cut stroke (left) to generate cross section on which X-ray CT image is visualized (right).

Since we have X-ray CT volumes, it is possible to provide a cross-section visualization. Fig 15 and S1 Video show our 3D model browser. It allows the user to browse a target model by interactively modifying the camera position with a mouse. It also allows the user to draw a mouse stroke to cut the model and generate a cross section, on which an X-ray CT image is visualized. With this application, it is possible to observe both naturally colored surfaces and internal structures. We believe that this visualization enhances the understanding of internal structures of specimens and is well suited for education.

### Comparison with existing digitization methods

**Fig 16:**
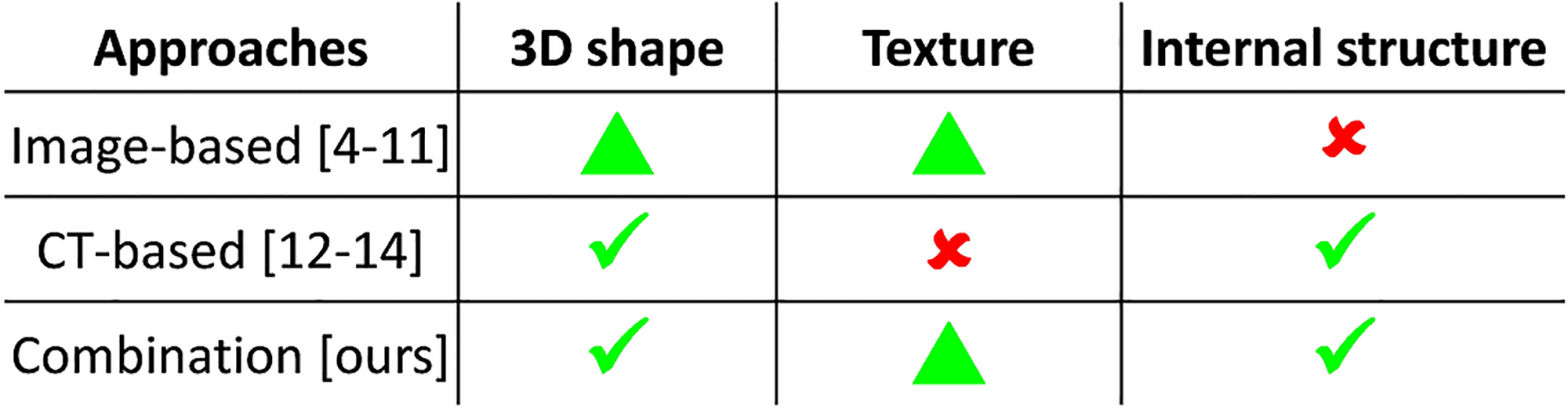
Capabilities of existing 3D digitization methods. Green triangle indicates limitations in reconstructing shapes and textures in occluded areas.

Existing measurement-based 3D digitization methods for insects and plants can be roughly classified into image-based [4–11] and CT-based [12–14] approaches. To clarify the advantage of our approach, we summarize the capabilities of each approach in Fig 16. The image-based approach [4–11] reconstructs 3D shapes from multiple photographs. Although this approach is able to generate shapes and textures at the same time, it does not capture internal structures. In addition, it misses the shapes and textures of the occluded areas of target specimens since a photograph does not capture areas being occluded. The CT-based approach [12–14] reconstructs a shape by segmenting a CT volume. Although this approach reconstructs a highly accurate 3D shape and captures internal structures, it does not measure surface textures. By combining both approaches, our proposed technique reconstructs 3D shapes and surface textures at the same time.

### Limitations and future work

As shown in Fig 16, our technique is still limited in reconstructing textures of occluded areas. Although we presented texture synthesis, it generates only a fake appearance. Developing a measurement-based approach for occluded areas to generate true textures remains as our future work.

**Fig 17:**
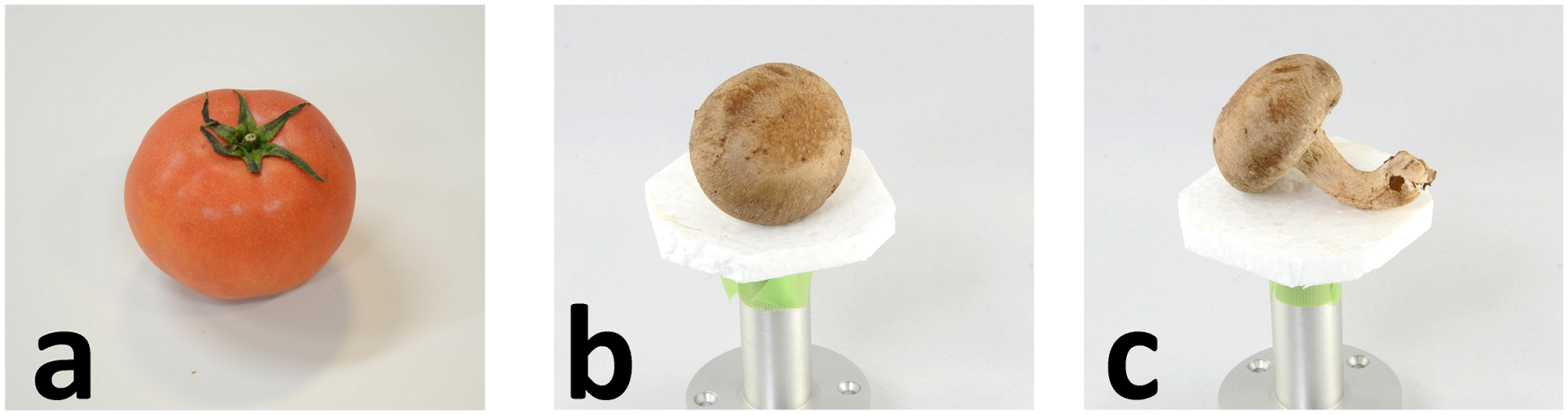
Photographs of highly symmetric objects. Our camera estimation fails for photographs (a, b) since their silhouettes match multiple viewpoints of target objects. It works well for photograph (c) with silhouette that matches unique viewpoint of target.

Another limitation of our technique is highly symmetrical objects. Since we estimate the relative camera position only from silhouettes, it is difficult to deal with a photograph of which the silhouette matches multiple viewpoints of a target object. Fig 17a and 17b show example photographs. Because a tomato and a Lentinula provide circle-like silhouettes when viewed from different points, our technique fails to accurately estimate the camera positions of these photographs. To deal with such objects, one solution is to record relative camera positions between photographs. For instance, the Lentinula has an asymmetric silhouette depending on the viewpoint, as shown in Fig 17c, and our technique works well for such photographs. If we recode relative (physical) camera positions between Fig 17b and 17c during photography, we can guess the camera position for Fig 17b by using the estimated camera position of Fig 17c. Furthermore, by using the relative camera positions of photographs, it would be possible to estimate all camera positions relative to a 3D model simultaneously, resulting in accurate calibration for symmetric objects. Another solution is to develop a marker that can be captured by both X-ray CT and digital cameras. We would like to work on these two solutions in the future.

Our current technique still requires user operation for CT volume segmentation, photograph segmentation, and taking photographs. Such operation would become a bottleneck when generating a huge digitization database. Our on-going future work is to automate the process completely.

## Conclusions

In this paper, we presented a technique for digitizing natural objects, such as insects and flowers. The key idea is to combine X-ray CT scans and photographs; we segment a CT volume to reconstruct a 3D shape model and back project photographs to the model to obtain its texture. We presented a technique for estimating the relative camera positions for each input photograph by using the silhouette information. We also provided a technique for merging multiple textures obtained from photographs taken from different viewpoints by extending graph-cut textures. We adopted a texture synthesis technique to surface model to retrieve occluded areas. We illustrated the feasibility of the presented technique by adopting it to flowers, insects, and mushrooms to create 3D textured models of them.

## Supporting information

S1 Video Supporting video. This video shows a rendered scene including nine models generated with our system and our cross section visualization tool.
(MP4)
